# Advances in the multimodal analysis of the 3D chromatin structure and gene regulation

**DOI:** 10.1038/s12276-024-01246-7

**Published:** 2024-04-25

**Authors:** Man-Hyuk Han, Jihyun Park, Minhee Park

**Affiliations:** 1 Department of Biological Sciences, Korea Advanced Institute of Science and Technology (KAIST), Daejeon, 34141 Republic of Korea; 2 Graduate School of Engineering Biology, Korea Advanced Institute of Science and Technology (KAIST), Daejeon, 34141 Republic of Korea; 3 KAIST Institute for BioCentury, Korea Advanced Institute of Science and Technology (KAIST), Daejeon, 34141 Republic of Korea; 4 KAIST Stem Cell Center, Korea Advanced Institute of Science and Technology (KAIST), Daejeon, 34141 Republic of Korea

**Keywords:** Chromatin structure, Transcription

## Abstract

Recent studies have demonstrated that the three-dimensional conformation of the chromatin plays a crucial role in gene regulation, with aberrations potentially leading to various diseases. Advanced methodologies have revealed a link between the chromatin conformation and biological function. This review divides these methodologies into sequencing-based and imaging-based methodologies, tracing their development over time. We particularly highlight innovative techniques that facilitate the simultaneous mapping of RNAs, histone modifications, and proteins within the context of the 3D architecture of chromatin. This multimodal integration substantially improves our ability to establish a robust connection between the spatial arrangement of molecular components in the nucleus and their functional roles. Achieving a comprehensive understanding of gene regulation requires capturing diverse data modalities within individual cells, enabling the direct inference of functional relationships between these components. In this context, imaging-based technologies have emerged as an especially promising approach for gathering spatial information across multiple components in the same cell.

## Mapping chromatin 3D structure: innovations in multimodal integration

Understanding how genes work and interact within our cells is key to understanding biology and disease. This review looks at the latest methods for analyzing the 3D structure of our genome, focusing on sequencing, and microscopy techniques. It shows how these technologies let us see our DNA structure in 3D and understand how this affects gene activity and cell functions. The authors aim to provide a fuller picture of how DNA’s spatial arrangement affects its function by combining different methods. They discuss methods that can concurrently map the landscape of chromatin structure along with the transcriptomic, proteomic, and epigenetic landscape, all critical for biological functions. Such a comprehensive perspective is vital for accurately unraveling the complex interactions and regulatory mechanisms within the nucleus, thereby enhancing our understanding of the functional significance of 3D chromatin architecture.

This summary was initially drafted using artificial intelligence, then revised and fact-checked by the author.

## Introduction

Deciphering the mechanisms of gene expression and regulation has been a cornerstone of molecular biology, with particular interest in the intricate interactions between genes and regulatory elements. Despite significant advances in sequencing and screening technologies, which have revealed a vast network of genes and regulatory elements, the complex and dynamic nature of their interactions continues to pose substantial challenges to fully elucidating their functions^1–4^.

Recent studies have underscored the critical role of the chromatin structure in gene regulation^5–10^, with techniques such as chromosome conformation capture (3 C) and its variants illuminating topologically associating domains (TADs) as fundamental units of the nuclear architecture^11^. These domains play a key role in gene regulation, and their disruption is linked to various developmental diseases and cancers^5,6,8,12^. Based on this understanding, the development and refinement of methods to dissect the three-dimensional (3D) architecture of the chromatin have become pivotal, as they enable detailed investigations into how the spatial organization of the chromatin influences its regulatory functions. To this end, a significant array of techniques have emerged, which are broadly categorized into sequencing-based and imaging-based approaches^13^. Sequencing-based methods, leveraging the power of next-generation sequencing and benefiting from the reduced costs and enhanced capabilities of modern sequencing technologies, map chromatin interactions on a genomic scale by identifying the frequency of close interactions. Alternatively, imaging-based methods, including DNA fluorescence in situ hybridization (FISH), utilize sequence-specific probes to capture the spatial arrangement of chromatin loci within the nucleus, offering high-resolution insights at the single-cell level.

While these technological advances have significantly enhanced our understanding of the three-dimensional conformation of the chromatin, it has become increasingly clear that insights derived solely from structural information fail to fully explain the functional roles of chromatin folding in regulating nuclear activities. To bridge this gap and unravel the structure‒function relationship in the genome, there is a compelling need for an integrative approach that employs multimodal techniques. These techniques should be capable of concurrently mapping the landscape of the chromatin structure, as well as the transcriptome^14–16^ and proteome^17–19^, both of which are pivotal for biological functions. Such a comprehensive view is essential for accurately deciphering the multifaceted interactions and regulatory mechanisms at play within the nucleus, thereby providing a more complete understanding of the functional significance of the 3D chromatin architecture.

In this review, we explore methods for analyzing the 3D organization of the genome, highlighting both sequencing- and microscopy-based techniques. We also highlight how these technologies not only enhance the visualization of the 3D chromatin architecture but also facilitate the capture of spatial information concerning key biological factors, including proteins, RNA, DNA, and chromatin modifications. These techniques aim to establish a more definitive link between the structure and biological function.

### Impacts of Hi-C technology and its evolving variants

**Fig. 1:**
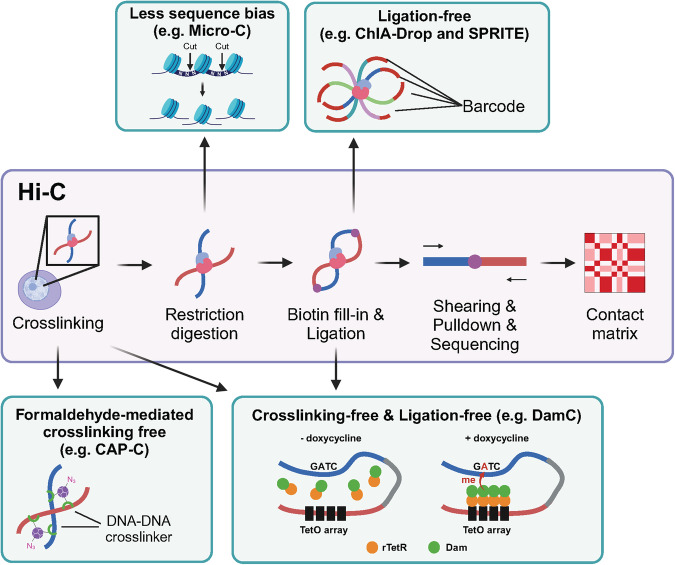
Overview of Hi-C and technologies overcoming its limitations. The Hi-C technique begins with the fixation of chromatin, with crosslinking preserving the proximity between interacting loci within lysed cells. The DNA is then digested using restriction enzymes, followed by the incorporation of biotinylated nucleotides at the cleavage sites. Subsequent ligation results in the formation of chimeric DNA fragments, which are then sequenced to identify locus interactions. The frequency of these interactions is represented in a 2D contact matrix, with matrix elements reflecting the interaction rates between pairs of genomic loci. To address the limitations of Hi-C, several technologies have emerged. To alleviate the interference from protein‒DNA crosslinking, certain methodologies (e.g., CAP-C) utilize DNA‒DNA crosslinkers. Additionally, approaches such as Micro-C employ DNase enzymes to minimize sequence bias. Some techniques (e.g., ChIA-DROP and SPRITE) have replaced ligation with barcode tagging to capture multiway interactions. Finally, Dam-based technologies (e.g., DamC) exploit Dam methyltransferase to capture in vivo interactions, bypassing crosslinking and ligation steps.

Advancements in our understanding of the 3D chromatin structure have been made by the advent of Hi-C technology^20^. This technique, based on proximity ligation and subsequent deep sequencing, is extensively utilized to delineate the chromatin architecture, offering insights into the frequency of contacts between chromatin regions and facilitating the creation of genome-wide interaction maps **(**Fig. 1**)**^20^. Hi-C has supported the concept of nuclear territories by identifying compartmentalized regions of active and inactive chromatin and has provided evidence for the hierarchical organization of these domains, which aligns with the previously theorized fractal globule model of unknotted, self-similar, and highly compartmentalized chromatin^21–23^. Within these compartments, Hi-C revealed a dominance of interactions among similar types (homotypic interactions), with the heterochromatin generally localized at the nuclear periphery. A more granular structure, known as TADs, with interactions within a TAD being more prevalent than those between TADs, has also been characterized. Hi-C has shed light on the complex interplay between the chromatin structure and gene regulation, revealing how changes in the 3D architecture can be linked to various genetic disorders, including F-syndrome, polydactyly, and brachydactyly^8^.

Nonetheless, Hi-C has several limitations^24,25^. Hi-C typically uses formaldehyde for crosslinking, which can impede the access of restriction enzymes to DNA owing to the nonspecific crosslinking of proteins, thereby affecting the resolution and signal-to-noise ratio. Additionally, the use of restriction enzymes introduces sequence bias, as they target specific DNA sequences. Finally, traditional ligation reveals only pairwise interactions and does not account for the multiway interactions occurring within the nucleus. To overcome these limitations, new derivatives of Hi-C have been developed that do not rely on crosslinking, restriction enzymes, or ligation (Fig. 1, Table 1).

**Table 1:** Representative technologies for the analysis of chromatin’s 3D architecture.

Name	Multi-modality	Advantages	Citation
Sequencing-based			
***Hi-C-based***			
Hi-C	DNA	- investigates the 3D architecture of whole genome	20-23
CAP-C	DNA	- improves signal-to-noise ratio	26
Micro-C	DNA	- improves resolution up to nucleosome level	27,28
ChIA-PET	DNA and Protein	- visualizes genome-wide interactions between chromatin and specific proteins	42,43,46
HiChIP	DNA and Protein	- visualizes genome-wide interactions between chromatin and specific proteins	40,44,45
HiChIRP	DNA and RNA	- can detect chromatin conformation associated with RNA of low amount	55
GRID-seq	DNA and RNA	- offer genome-wide DNA-RNA interactions	50
MARGI	DNA and RNA		49
ChAR-seq	DNA and RNA		51
***Non-Hi-C-based***			
ChIA-Drop	DNA and Protein	- needs fewer samples for investigation - provides multi-way interactions - capture single-molecule level interactions	31,32
DamC	DNA	- captures in vivo chromatin conformation without crosslinking and ligation	34
SPRITE	DNA and RNA	- provides multi-way interactions - is able to capture broad range of confirmation from loops to interchromosomal interactions	32,33,56
**Imaging-based**			
***Volumetric chromatin tracing***			
OligoSTORM	DNA	- provides detailed 3D structure and spatial location - highly compatible with other modality imaging	68-71,83,85,87
OligoDNA-PAINT			70,81,82,84,86
***Polymeric chromatin tracing***			
ORCA	DNA, RNA, protein	- provides 3D chromatin structure and high-throughput multimodal information	59
Hi-M			64
MINA			67
DNA seqFISH+			57,58,73

To capture chromatin conformation along with other biological modalities such as RNA and protein, numerous technologies have rapidly emerged. These can be classified into sequencing-based and imaging-based technologies. Sequencing-based technologies further divide into Hi-C-based technologies, which utilize proximity ligation, and non-Hi-C-based technologies. Thanks to advancements in FISH methods, imaging-based technologies have evolved at an accelerated pace. These can be subdivided into volumetric technologies, capturing the volumetric space of the target genomic region, and polymeric tracing technologies, pinpointing the centroid of the target genomic region. Advanced imaging-based technologies are also capable of capturing a range of biological modalities. Many imaging technologies lack designated names and are therefore omitted from this table.

First, You et al. introduced a method called chemical-crosslinking assisted proximity capture (CAP-C)^26^, which circumvents the biases from protein‒DNA crosslinking. CAP-C employs multifunctional poly(amidoamine) dendrimers and UV irradiation to covalently bind dendrimers to DNA fragments, facilitating the removal of DNA-bound proteins. This results in consistent DNA fragmentation, with fragments ranging from 50 to 200 base pairs, reducing the background noise and improving precision and sensitivity. Consequently, CAP-C allows for the high-resolution detection of transcription-dependent changes in multiple layers of the chromatin conformation, such as loops, domains, and subnuclear compartments, strengthening the link between transcription and the chromatin organization. This research demonstrated that transcription, specifically the initiation of transcription, primarily influenced local chromatin organization on a short scale and that inhibiting transcription led to reduced chromatin interactions.

Second, Hsieh et al. developed Micro-C, which bypasses the sequence specificity of restriction enzymes by utilizing micrococcal nuclease (MNase), which cleaves DNA in nucleosome linker regions^27^. This method enables the generation of chromosomal folding maps with a higher resolution by capturing a more detailed chromatin conformation without the need for additional enrichment processes that are required by Hi-C, such as protein-centric or promoter-centric capturing techniques^28–30^. For instance, Micro-C analysis in budding yeast revealed self-associating domains that were considerably smaller than TADs and were previously undetected by other methods because of resolution limitations; this contributed to revealing the molecular mechanisms underlying chromosome compaction at the nucleosome level.

Finally, Zheng et al. developed chromatin interaction analysis via droplet-based and barcode-linked sequencing (ChIA-Drop), a ligation-free technique that detects complex chromatin interactions^31^. ChIA-Drop combines chromatin immunoprecipitation (ChIP) with DNA barcoding to identify interactions involving specific proteins. This method can reveal more complex interactions per specific site that cannot be detected by paired ligation technologies. This study characterized transcriptional multiplex interactions and found that the majority of active chromatin complexes involved only one promoter interacting with distal nonpromoter elements, challenging previous analyses that indicated widespread promoter–promoter interactions.

As such, this pairwise ligation-free approach has the potential to reveal sophisticated landscapes involving genes such as developmental genes^32^. Moreover, split-pool recognition of interactions by tag extension (SPRITE), which was developed by Quinodoz et al., forgoes ligation in favor of repeated split-pool tagging, in which each molecule in an interacting complex contains a unique series of concatenated barcodes^33^. SPRITE can simultaneously capture a broad spectrum of interactions, from consecutive loops to interchromosomal interactions^32,33^. This approach provided a picture in which different nuclear bodies had different transcriptional activities, and even genes on different chromosomes belonged to the same nuclear body.

Another technique, DamC, which bypasses both crosslinking and ligation, builds upon the DNA adenine methyltransferase identification (DamID) technique, which was originally devised for studying protein‒DNA interactions in vivo. DamID utilizes Dam methyltransferase to tag DNA at protein-binding sites with adenine methylation. The methylated DNA fragments are then sequenced, allowing DamID to effectively identify DNA‒protein interactions in a crosslinking-free manner, thereby preserving their native cellular context. However, DamID lacks formal schemes for calculating contact probabilities, which prevents the detection of TAD boundaries and CTCF loops. As such, DamC provides a physical model of methylation dynamics that calculates contact probabilities, thereby generating contact frequency maps from methylation state results. DamC utilizes the integration of TetO arrays, which allows for the recruitment of rTetR fused with Dam to the targeted site, offering a 4C-like viewpoint. This approach not only validates the presence of TADs and CTCF loops in vivo but also demonstrates that the frequency maps produced by DamC align with those obtained from Hi-C, supporting the accuracy of biophysical models based on Hi-C data^34^.

This progression enhances our understanding of the intricacies of the 3D chromatin conformation. However, to establish a more definitive link between the chromatin structure and biological function, sequencing-based technologies are evolving to capture spatial relationships not only between DNA but also between DNA and other molecules, including RNA and proteins, and epigenetic features. In the next section, we discuss how sequencing-based multiomics and combinatorial analyses are being used to investigate these complex relationships.

### Sequencing-based technologies for integrative views of genomic regulation in three dimensions

Deciphering the network of protein‒DNA interactions is crucial for understanding transcriptional regulation and many other biological activities. Identifying where transcription factors and other DNA-binding proteins attach to the genome helps unravel this complex process. Integrating the 3D structure of the chromatin with detailed maps of protein binding and epigenetic modifications is now recognized to be essential for a full understanding of gene regulation.

The associations between proteins and DNA were initially mapped using pulldown and sequencing techniques, which are used to isolate DNA-binding proteins along with their bound DNA for joint analysis^35–38^. Chromatin immunoprecipitation sequencing (ChIP-seq) has been pivotal as it enables genome-wide mapping of chromatin marks and protein-binding sites. This method initially provided one-dimensional views of epigenetic or protein-binding profiles of the genome. Further advances have since extended the utility of ChIP to capture the three-dimensional context of these associations^39–42^. Fullwood et al. pioneered chromatin interaction analysis with paired-end tag sequencing (ChIA-PET), in which sonicated chromatin–protein complexes are enriched by ChIP and DNA fragments are ligated based on proximity^42^. In this scheme, remote chromosomal regions, which are brought together into close spatial proximity by protein factors, are ligated and sequenced together, enabling the elucidation of the 3D genome-wide interactome involving specific proteins, such as estrogen receptor α (ER-α). Furthermore, Mumbach et al. introduced HiChIP, a method similar to ChIA-PET but with an improved yield of conformation-informative reads and a greater efficiency regarding the amount of the input required compared with that in ChIA-PET^40^. Long-range DNA contacts are first established in situ within the nucleus, followed by ChIP, which facilitates the targeted capture of long-range chromatin interactions associated with specific proteins. By reversing the sequence of proximity ligation and ChIP, HiChIP enhances the signal-to-noise ratio and operates efficiently with less starting material than that required for ChIA-PET^40^. ChIA-Drop can also specifically capture chromatin conformations involved in specific protein complexes that are enriched by ChIP. Leveraging droplet-based techniques, ChIA-Drop enables the capture of single-molecule interactions between proteins and DNA^31^. These techniques have enabled the mapping of functionally relevant interactions between target genes and transcription factors on a genome-wide scale^43^. Targeting key proteins, such as RNA polymerase II and CTCF, by HiChIP has proven effective in identifying promoter-centric interactions, which highlights the utility of this technique in revealing significant structural contributions to biological functions^44,45^. These techniques have provided insights into the molecular underpinnings of various diseases, including cancer^42^ and blood disorders^46^.

In addition to proteins, nuclear RNA has been suggested to be a structural component of the nuclear matrix, potentially organizing the higher-order structure of chromatin^47^. Recent techniques have been developed to discover global interactions between RNA and the genome and to explore the role of RNA in chromatin organization^48^. MARGI, GRID-seq, and CHAR-seq allow the capture of de novo interactions between DNA and RNA by utilizing a bivalent linker. This bivalent linker addresses the limitations on the RNA target range that arise from the necessity of using complementary probes in previous techniques for examining RNA and DNA interactions^49–51^. The identification of these de novo DNA‒RNA interactions offers valuable resources for future research^52–54^. Additionally, findings from these technologies reinforce the connection between RNA and gene regulation, showing a positive correlation between RNA attachment and active histone marks at promoters when DNA–RNA interaction data are compared with ChIP–seq data^49^. These findings also demonstrate the association of tissue-specific RNA with active promoters and enhancers^50^. However, these techniques face challenges in detecting low-abundance RNAs and complex, multiway interactions.

Long noncoding RNAs (lncRNAs) have become recognized as key players in the organization of chromatin structure, but their typically low expression levels complicate the study of their interactions with chromatin. To address this issue, Mumbach et al. developed high-throughput chromatin isolation by RNA purification (HiChIRP), a method that enriches a specific RNA, notably enhancing the detection of nuclear RNA interactions, even at levels nearly as low as ten copies per cell^55^. The HiChIRP technique enhances RNA capture in chromatin conformation studies by incorporating azido-modified nucleotides during 3 C to enrich RNA with biotinylated probes and using dibenzocyclooctyne (DIBO) “click” chemistry to covalently attach biotin for chromatin contact enrichment. This technique revealed, for example, that long intergenic noncoding RNA (lincRNA)-EPS was significantly involved in interactions at CTCF boundaries and promoter regions, highlighting the role of RNA in complex genomic interactions.

In addition, Quinodoz et al. further advanced our understanding of DNA–RNA interactions with the development of RNA & DNA SPRITE (RD-SPRITE)^56^. This advanced version of SPRITE^33^, featuring increased RNA-tagging efficiency, facilitates the simultaneous high-resolution mapping of thousands of RNAs relative to all other RNA and DNA molecules in a 3D space. Because of the ability to detect low-abundance RNAs, such as individual nascent pre-mRNAs and ncRNAs, this approach has revealed numerous higher-order RNA–chromatin hubs and territories rich in noncoding RNAs (ncRNAs). Additionally, perturbation experiments revealed that the absence of ncRNAs destroyed corresponding nuclear hubs, underscoring the role of RNA in the formation of functional nuclear hubs. These findings emphasize the pivotal roles of RNAs in key nuclear processes, such as RNA processing, heterochromatin assembly, and gene regulation, by influencing long-range DNA contacts and recruiting ncRNAs and protein regulators within these territories.

To unravel the structure–function relationships of genomes and link specific 3D conformations to nuclear processes, numerous methodologies have been developed to capture DNA interactions with other molecules, such as RNA and proteins. However, emerging evidence from using various single-cell techniques (e.g., scHi-C, scSPRITE, and imaging approaches) highlights a significant cell-to-cell heterogeneity in genome organization, underscoring the need for methods that can simultaneously probe the chromatin structure, RNA, and proteins within the same single cells^57–62^. Such comprehensive analysis is crucial for establishing a more definitive causal link between structure and function. While sequencing approaches such as ChIA-Drop offer avenues for concurrent single-cell analysis, imaging-based methods present a valuable complementary strategy. These methods can not only visualize the 3D positions of specific genomic loci at the single-cell level but also have the potential to be adapted for the efficient detection of other components, including RNA and proteins. This capability can significantly enrich our understanding of the intricate web of genomic interactions.

### Visualizing the genomic landscape with chromatin tracing

Imaging technologies, notably FISH-omics, have significantly enhanced our capability to directly explore chromatin structures, with an inherent advantage of enabling single-cell visualization^63^. These methods facilitate the mapping of 3D coordinates across vast genomic regions along a single chromosome, ranging from hundreds of kilobases^59,64,65^ to several megabases^61,62,66,67^, and even the entire genome^57,58^. Traditional DNA FISH offers a diffraction-limited view of targeted genomic regions but fails to delineate complex chromatin folding paths because simultaneous visualization of many genomic loci with similar fluorescence colors obscures their individual identities.

**Fig. 2:**
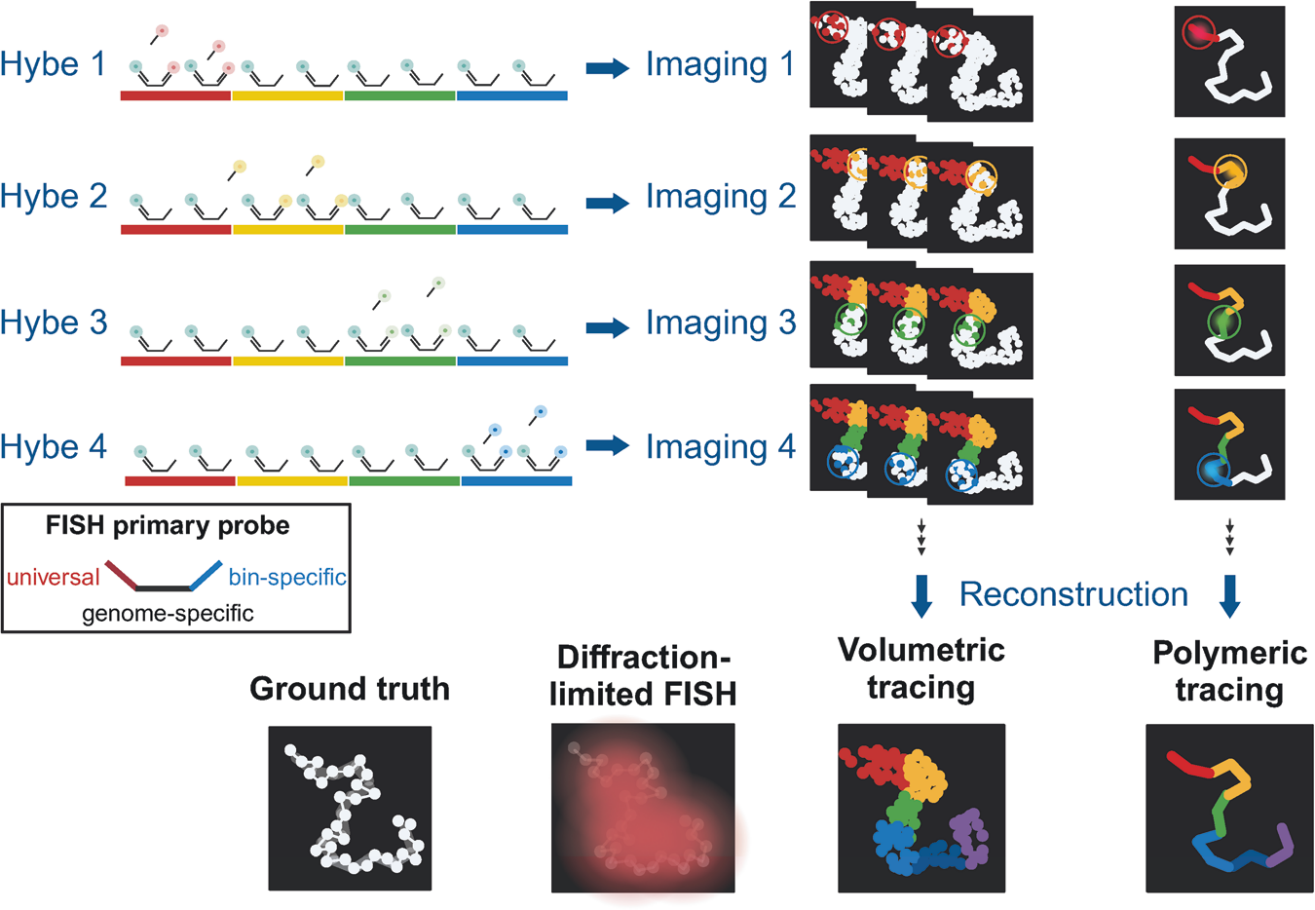
Exploration of the 3D chromatin architecture through chromatin tracing. Chromatin tracing is an imaging-based methodology used to reconstruct the 3D structure of specific chromatin regions. This technique involves segmenting the target chromatin into bins and employing a combination of FISH probes. These probes are designed to target “genome-complement”, “universal”, and “bin-specific” sequences. Dye-conjugated adapters, which are complementary to each bin-specific sequence, are sequentially hybridized and visualized via microscopy. Two primary approaches that are utilized in chromatin tracing are volumetric and polymeric tracing. Volumetric tracing combines stochastic illumination with subpixel localization techniques to generate superresolution density maps for each genomic bin. Polymeric tracing, on the other hand, involves simultaneous illumination of dyes within a single genomic bin. The centroid of each bin is used to reconstruct the 3D structure of the chromatin.

However, advancements in superresolution microscopy^68,69^ and the development of high-throughput methods for single-stranded oligonucleotide synthesis, such as Oligopaint library approaches^70,71^, have overcome these limitations. This superresolution is achieved by segmenting genomic regions into smaller sections and sequentially imaging them, which allows “walking” along whole chromosomes and provides a continuous polymeric view of individual chromosome trajectories, often referred to as “chromatin tracing” techniques^57–61,64,65,67,72–77^. Chromatin tracing involves a sequential process of hybridization and imaging, followed by computational reconstruction. It starts by binding a collection of primary FISH probes to the entire target genomic site, with every short segment of chromatin receiving its own barcode. Subsequently, secondary FISH probes, each marked with a fluorescent tag, are sequentially attached to unique tail regions of the primary probes, which are specific to each genomic site. This method distinguishes each barcoded region as a distinct, diffraction-limited spot in three dimensions, allowing for the precise determination of its center (i.e., x, y, and z coordinates) with a nanometer accuracy. Before initiating the hybridization cycle for the subsequent barcode region, the fluorescence signal from each barcode site is extinguished using methods such as photobleaching, fluorophore cleavage, or strand displacement^59,62,65,78^. Ultimately, by linking the central positions of these imaged genomic sites based on their genetic identities, a detailed 3D path of the chromatin folding landscape can be constructed, providing unparalleled insights into the spatial organization of the genome at the single-cell level (Fig. 2, Table 1).

Chromatin tracing employs two main approaches, namely, volumetric^61,70,79–87^ and polymeric^58,59,64–67^ approaches **(**Fig. 2**)**. Volumetric chromatin tracing techniques, such as Oligopaint stochastic optical reconstruction microscopy (OligoSTORM) and Oligopaint DNA-based point accumulation for imaging in nanoscale topography (OligoDNA-PAINT), integrate Oligopaint technology for FISH with STORM and DNA-PAINT for imaging, respectively, to enable in situ single-molecule superresolution imaging of nucleic acids. Volumetric chromatin tracing reveals density clouds of target structures, typically used to understand chromatin compaction and asphericity^61^ or a spatial overlap between neighboring gene domains^80^. In contrast, polymeric chromatin tracing identifies the centroid of an imaged spot as representing the entire targeted genomic segment during each hybridization cycle. A 3D chromatin folding model is then constructed by sequentially connecting the centroids of spots imaged in each round. This approach correlates genomic segments within targeted chromatin directly with genomic bins via 3 C methods, revealing significant similarities in the chromatin structure at the population level, as demonstrated by optical reconstruction of chromatin architecture (ORCA), high-throughput, high-resolution, high-coverage, microscopy-based (Hi-M), and multiplexed imaging of nucleome architectures (MINA) methods^59,62,64,67^.

Chromatin tracing techniques have provided pivotal insights into the complex architecture of the genome^59,61,64,65,67,72,88^. These methods have been applied to a range of genomic scales and cell types, as well as to model organisms, from *Drosophila* embryos^59,64^ to *Caenorhabditis elegans*^88^ and mammalian systems^61,62,67^. Key discoveries include mapping the spatial dynamics of enhancer–promoter interactions^59,64^ and unraveling the structural reconfigurations within TADs and A/B compartments in response to transcriptional changes^66,67^. These advances underscore the potential of microscopy-based imaging to offer a granular view of chromatin organization, shedding light on the underlying biological processes and the mechanisms involved.

### Receptivity of imaging-based techniques to multimodality

The significance of spatial interactions of genomic elements is increasingly recognized. Although sequencing-based technologies have advanced our understanding of genome organization, they may not fully capture the spatial context as they require dissociating cells. Additionally, integrating new modalities into sequencing-based approaches can be challenging because of the limited number of reads available per cell. Imaging-based technologies, on the other hand, offer a complementary approach by directly visualizing the spatial distribution of genomic elements (DNA, RNA, and proteins), which enables comprehensive multimodal analysis and high-throughput data generation.

**Fig. 3:**
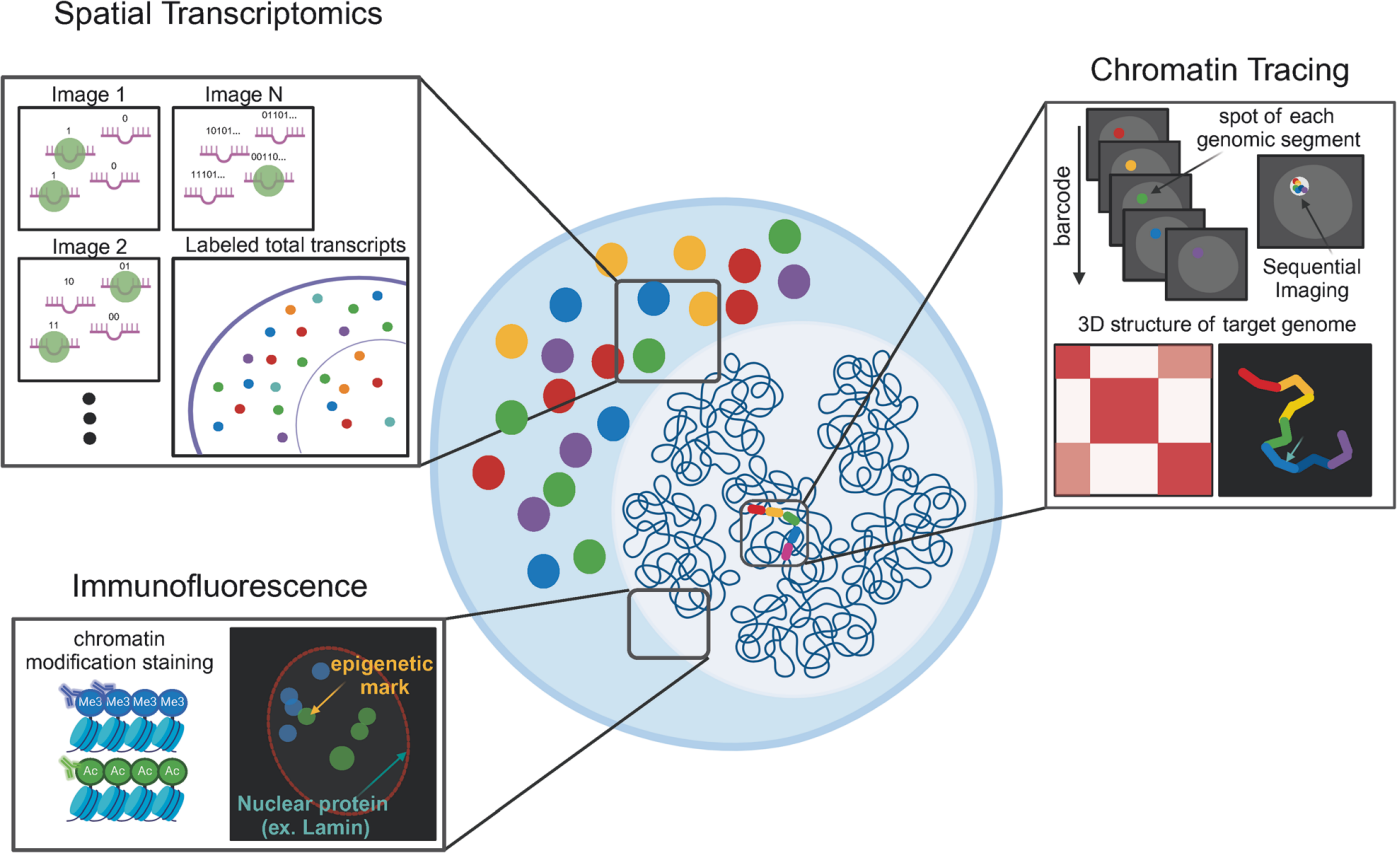
Single-cell multimodal imaging. The integration of multiplexed single-cell FISH with spatial transcriptomics and protein localization unravels the complex layers of cellular information. In spatial transcriptomics, individual RNA molecules are tagged with unique barcodes through successive rounds of hybridization and imaging to capture gene expression levels while preserving spatial distribution data. Immunofluorescence is used to label nuclear proteins or chromatin marks to visualize their precise positions within the cell nucleus. Chromatin tracing allows the mapping of the 3D structure of chromatin by targeting specific genomic segments and determining their centroids to reconstruct the three-dimensional folding pattern of the chromatin. Together, these imaging techniques provide a comprehensive view of the functional architecture of cells at the single-cell level.

Spatial multimodal imaging represents a strength of imaging-based approaches^72^ (Fig. 3). Oligopaint technology has played a crucial role in this respect, enabling the generation of a sufficient number of oligonucleotides to label thousands of transcripts alongside genomic segments^57,58,74,76,77^. Complex barcoding and superresolution imaging techniques make it possible to simultaneously localize thousands of transcripts, with precise spatial information. This multimodal approach extends beyond transcriptomics^57–60,73^, allowing for the visualization of histone modifications^57,65^ and associations with the nuclear lamina and nucleolar structures^66,67^, concurrently with chromatin tracing.

For example, Mateo et al. developed the optical reconstruction of chromatin architecture (ORCA) to combine the transcriptomes of approximately 30 RNA species with the 3D configuration of the Hox gene cluster in *Drosophila* embryos. They discovered that RNA expression patterns and the physical boundaries between active and polycomb-repressed DNA are intricately linked, such that spatial interactions within the 3D chromatin structure can enhance the RNA expression of a cell. Adding a new modality to the experiment can be easily achieved by constructing a new oligo set targeting additional RNA or DNA sequences. For more high-throughput transcriptome imaging, multiplexed error-robust FISH (MERFISH) allows for the spatial distribution of hundreds or thousands of RNA species to be elucidated. Each RNA species is labeled with approximately 192 encoding probes, transforming the RNA into a unique combination of readout sequences. This process enables the decoding of RNA species through sequential hybridization and imaging^76^. Su et al. integrated MERFISH with multiscale, multiplexed chromatin tracing to achieve simultaneous imaging of more than 1,000 genomic loci and nascent transcripts of more than 1,000 genes, together with landmark nuclear structures, including nuclear speckles and nucleoli^66^. They observed a correlation, at the single-cell level, between chromatin compartments and the local transcription levels, as well as the dynamic transition of *trans-*chromatin interaction hubs between the lamina and nuclear speckle.

Multimodal imaging extends beyond integrating spatial transcriptomes with chromatin tracing. Proteins, nuclear compartments, and histone marks can also be targeted in a semihigh-throughput manner by conjugating oligonucleotides to antibodies to enable the selective readout of individual primary antibodies with fluorescently labeled readout FISH probes^73^. Notably, Takei et al. conducted seqFISH+ in which they examined chromosome structures, nuclear bodies, chromatin states, and gene expression within individual cells^58^. They imaged 3,660 chromosomal loci alongside 17 chromatin marks and 70 RNAs. Such multimodal imaging provides a direct representation of the functional relevance of the 3D configuration of chromatin. Their findings indicate that numerous DNA loci, particularly those associated with active genes, tend to be located on the periphery of nuclear bodies and at boundary interfaces. This spatial arrangement suggests that regulatory factors might navigate in a two-dimensional manner along the surface of these interfaces to locate their target genes more efficiently. Additionally, they observed that stable chromatin states were inherited over several generations, which implies their possible functional role in gene regulation^58^. In another single-cell, high-resolution, multimodal imaging study, researchers analyzed subnuclear compartments, associated genomic loci, and their impacts on gene regulation directly within individual cells in the adult mouse cerebellum. This two-layer barcoding DNA seqFISH+ study showed that repressive chromatin compartments were more variable by cell type than active compartments and revealed cell type-specific enrichment of constitutive heterochromatin clusters at specific gene loci^57^. Furthermore, they found physical evidence that cell type-specific facultative and constitutive heterochromatin compartments were enriched at specific genes and gene clusters, shaping a radial configuration in neurons and glial cells.

Sequencing-based technologies, such as HiChIP and RD-SPRITE, offer insights into the spatial relationships between DNA and other molecular entities, such as proteins or RNA. However, imaging methods stand out for their single-cell resolution. Imaging-based approaches enable the simultaneous capture of spatial information from various modalities within individual cells, including the 3D configuration of DNA, spatial transcriptomics, and protein‒DNA interactions. This capability allows for the direct inference of causality at the single-cell level, a distinction not achievable with sequencing-based technologies, which mainly infer correlations. Multimodal high-throughput imaging provides a comprehensive view of chromatin organization in its native structural and functional contexts. We anticipate a widespread adoption of these advanced, high-throughput, and multimodal imaging technologies to facilitate the generation of a more accurate structure‒function atlas of the genome.

### Concluding remarks

The relationship between the 3D chromatin structure and its biological consequences continues to be a pivotal area of investigation. Progress in both sequencing-based and imaging-based techniques has significantly enhanced the resolution of visualization of three-dimensional chromatin structures. Furthermore, exploring the functional role of the 3D chromatin structure necessitates understanding the spatial relationships among various biological entities that interact with or are related to chromatin. Techniques are being refined to simultaneously visualize multiple modalities within the same cell. We suggest that imaging-based technologies offer significant advantages in this domain. High-throughput sequential FISH technologies facilitate a more ready adaptation to several modalities than do sequencing-based approaches. Moreover, the ability to visualize individual cells by using imaging-based technologies allows us to circumvent the challenges posed by the heterogeneity in capturing direct relationships.

Challenges still remain in capturing the dynamic nature of cellular processes over time. The temporal variations in the chromatin structure, such as those occurring during cell cycles^89^ or developmental stages^90^, introduce additional complexity to our understanding. Most current methods focus primarily on analyzing fixed cells. However, single-cell analyses, particularly scRNA-seq, have advanced in mapping cellular trajectories over pseudotime^91–94^. This approach models continuous transcriptomic changes as trajectories within a simplified, low-dimensional space. Although these pseudotime trajectories generally accurately mirror real differentiation pathways, the presence of subtle variations highlights the need for further refinement. The integration of single-cell imaging with complementary assays has emerged as a promising frontier, offering the potential for more precise temporal and structural transcriptomic profiling. The advent of such techniques holds the potential to revolutionize our temporal understanding of cellular function and chromatin dynamics.
